# Premenstrual Syndrome Is Associated with Dietary and Lifestyle Behaviors among University Students: A Cross-Sectional Study from Sharjah, UAE

**DOI:** 10.3390/nu11081939

**Published:** 2019-08-17

**Authors:** Mona S. Hashim, Asma A. Obaideen, Haitham A. Jahrami, Hadia Radwan, Hani J. Hamad, Alaa A. Owais, Lubna G. Alardah, Samir Qiblawi, Nabeel Al-Yateem, “Mo’ez Al-Islam” E. Faris

**Affiliations:** 1 Department of Clinical Nutrition and Dietetics, College of Health Sciences/Research Institute of Medical and Health Sciences (RIMHS), University of Sharjah, P.O. Box 27272, Sharjah, UAE; 2 Department of Clinical Nutrition, Faculty of Medicine and Health Sciences, University Putra Malaysia, 43400 Seri Kembangan, Malaysia; 3 Ministry of Health, Manama, Bahrain; 4 College of Medicine and Medical Sciences, Arabian Gulf University, Manama, Bahrain; 5 Department of Food Science and Nutrition, Faculty of Agriculture, Jerash University, Jerash 26150, Jordan; 6 Department of Histopathology, College of Medicine, Hail University, Hail 55462, Saudi Arabia; 7 Department of Nursing, College of Health Sciences/Research Institute for Medical and Health Sciences, University of Sharjah, P.O. Box 27272, Sharjah, UAE

**Keywords:** Premenstrual Syndrome (PMS), anthropometry, dietary habits, lifestyle behaviors

## Abstract

Premenstrual syndrome (PMS) is a cyclical late luteal phase disorder of the menstrual cycle whereby the daily functioning of women is affected by emotional and physical symptoms substantially interfering with their quality of life. Little is known about PMS in the United Arab Emirates (UAE). This study aimed to determine the prevalence and severity of PMS among university students in Sharjah, UAE, and clarify its associations with dietary habits, lifestyle behaviors, and anthropometric factors. A cross-sectional study was conducted on female college students at the University of Sharjah, UAE. Data were collected using self-administered questionnaires and anthropometric assessments. Descriptive statistics and multiple logistic regression analyses were performed. Participants were 300 adult university students aged 18–24 years (mean age 20.07 ± 1.53 years). In total, 95% of participants reported at least one PMS symptom during their menstrual period. The prevalence of PMS was 35.3%, with mild symptoms being the most commonly reported. Multiple regression analysis showed that smoking was associated with increased risk of reporting psychological (OR 2.5, 95% CI 1.1–5.8; *p* < 0.05) and behavioral symptoms (OR 2.2, 95% CI 1.0–4.9; *p* < 0.05), while high calorie/fat/sugar/salt foods intake was associated with increased risk of reporting physical symptoms (OR 3.2, 95% CI 1.4–7.3; *p* < 0.05). However, fruit consumption (OR 0.34, 95% CI 0.125–0.92; *p* < 0.05) was associated with a decreased risk of reporting behavioral symptoms. A high prevalence of PMS was reported among university students, with smoking and high calorie/fat/sugar/salt food consumption identified as strong risk factors for PMS.

## 1. Introduction

Premenstrual syndrome (PMS) is a cyclical late luteal phase disorder of the menstrual cycle whereby the daily functioning of women is affected by emotional and physical symptoms substantially interfering with her quality of life [1,2]. This syndrome is presented in a combination of symptoms that are characterized by physical, behavioral, and psychological changes, which some women experience from a week before to a few days into menstruation. The intensity of PMS varies among women according to hormonal, psychosocial, and physiological factors [3]. PMS has been reported to be a limitation for female adolescents and young adults at a time when they are aspiring to achieve developmental goals. PMS may lead to decreased occupational productivity, lower health-related quality of life, increased dependence on specialized healthcare, and interference with interpersonal relationships and daily living activities [4,5,6]. Further, PMS may increase the risk for hypertension [7,8], reduce the work-related quality of life [9], negatively impact the athletic performance and daily activities in collegiate athletes [10], and is significantly associated with academic performance impairment [11]. Further, alterations in cognitive–emotional processes have been reported to be associated with PMS [12].

Like many other syndromes, PMS is the output of the interaction between various genetic (presented in race/ethnicity) [13] and lifestyle behaviors [13,14,15,16], with dietary factors considered among the most influential [17]. The literature supports the association between cultural context/ethnicity and PMS and other premenstrual disorders, with different cultural contexts, thought to have different effects on the manifestations and consequences of PMS [18,19,20]{Davis, 2014 #61}. Cultural and ethnic manifestations for the way how girls deal with menstruation are reflected in the presence of certain beliefs, depicting menstruation as a hygienic crisis and as a secret event resulting in a culture of concealment surrounding menstruation, as reported for Egyptian adolescent girls [18]. Further, cultural manifestations are expressed in the different measures used by menstruating girls to alleviate menstrual pain, including the use of a heating pad, rest, herbal drinks, exercise and the use of non-contraceptive hormonal medications [18].

Female university students are among those most affected by PMS. The rate of PMS is known to be high among this group, and adversely affects their quality of life and academic performance [11,21,22,23,24,25,26,27,28,29]. The reported prevalence of PMS among female university students varies between different countries; for example, 33.82% in China [30], 37% in Ethiopia [11], 39.9% in Taiwan [22], 39.4%–56.9% in Iran [26,31], 65% in Egypt [18], 72.1%–91.8% in Turkey [6,21], 79% in Japan [23], 80% in Pakistan [25], 89.5% in South Korea [29], and 80.2%–92.3% in Jordan [27,28]. This geographical variability in the prevalence of PMS may be attributable to differences in genetic, dietary, and lifestyle factors among the young adult females examined. Differences in the prevalence of PMS may also be explained by community-adopted practices before and during menstruation [32], as well as differences in study methodology such as controls for confounding variables [33], methods of assessment, and independent variables. Further, variability in reported prevalence of PMS across cultures may also be accounted for by differences in the social meaning or construction of particular embodied and psychological experiences as a disorder associated with the reproductive body [34,35].

In the United Arab Emirates (UAE), Gulf Cooperation Council (GCC), and other Islamic countries, female adults have their own community and culture-acquired health beliefs, behaviors and practices with regard to puberty and menstruation [36,37,38,39], with various dietary and personal lifestyle behaviors and remedial approaches they follow to alleviate menstrual and premenstrual symptoms [40,41]. These approaches include the use of vitamins, following a healthy diet and using analgesics [40], personal hygiene, increased intake of vegetables and fruits, and use of exercise and warm showers [41].

In an earlier study among female school adolescents in UAE, it was reported that PMS was prevalent in this group and adversely affected their educational and social functions and emotional well-being, representing a significant public health problem [42]. However, no previous studies have investigated the prevalence of PMS and the association between PMS and dietary and lifestyle factors among university students in UAE. Thus, this study aimed to clarify the associations between PMS and dietary habits, lifestyle behaviors, and body composition variables as potential risk factors. 

## 2. Material and Methods

### 2.1. Design and Setting 

This quantitative cross-sectional study was conducted from February to May 2016 at the University of Sharjah (UOS), UAE, using self-administered, structured questionnaires. 

#### Research Consent and Permissions 

Approval for this study was obtained from the UOS Research Ethics Committee (REC/16/02/07/S) and official permission was taken from the dean of female students at the UOS. Participants were informed that the information collected would be kept with complete confidentiality and security, and would not be abused. For confidentiality purposes, names and addresses of participants were not collected. Participation was voluntary, and no monetary or non-monetary incentives were given. Participants received thorough verbal explanations about the study and its aims and objectives before providing written informed consent. Eligibility criteria were an adult female student registered at UOS for the current academic year, with regular menstrual periods (at least in the last three consecutive months), aged 18–24 years, willing to participate in the research, and willing to provide informed consent. Exclusion criteria were: pregnancy; known history of chronic illnesses such as sickle cell anemia, diabetes etc., or any psychiatric disorders; irregular menstrual cycles; and age younger than 18 years or older than 24 years. Even though oral contraceptives are among the factors that affect PMS prevalence and severity [43,44], questions regarding oral contraceptives were not raised as these tools are not commonly used because of students’ sociocultural traditions and religious beliefs [45]. Because the vast majority of the study participants were single (98%) and that sex before marriage is legally and culturally forbidden in Islamic and Arabic countries, including UAE, asking about using contraceptives among this group is considered sensitive and offensive.

### 2.2. Sample Size and Sampling Procedure

This cross-sectional study collected data from a convenience sample of 300 female students aged 18–24 years were recruited from different UOS colleges and campuses. We invited all female UOS college students (about 8710 students) to participate in the cross-sectional study. At least one reminder was sent to the students to participate, and the site of data collection was assigned in the invitation letter. Further, campaign for research participation was announced throughout the university website, social media (Facebook, Instagram, and Twitter), along with personal communication with female students in UOS. Those interested students who visited the assigned data collection site were met, and study objectives and protocol were elaborated before giving the consent form for signing their approval. Female students from different countries were recruited and included locals (Emirati), non-local GCC residents, Arab non GCC expatriates, as well as non-Arab non GCC residents. 

The sample size was determined using a single proportion for a finite population, assuming 30% non-response. Sample size calculations using Stata 13.1 with an alpha of 0.05 (two-sided) and 80% power (β = 0.2) indicated that a minimum of 150 participants was needed to determine a prevalence proportion of at least 30%. Furthermore, the sample size calculations took into consideration the issue of power for detecting associations between PMS and lifestyle and anthropometric factors. To estimate sample size a moderate effect size of approximately Cohen’s d of 0.5, the level of significance or type I error of 0.05 (5%), and a power or type II error or (1-β) of 0.8 (80%) were set. The resultant sample size calculations all suggested that a sample of 180–220 would be sufficient for performing the analyses. 

### 2.3. Data Collection Tools

A self-administered and semi-structured questionnaire was used for data collection. The questionnaire included pertinent demographic characteristics of the study participants, their obstetrics and gynecologic profiles and possible symptoms of PMS assumed to be developed that were collected from different kinds of literature. Pilot data testing was carried out, before starting the study, on 25 (8.3% of the targeted sample size of 300 female students) female students of UOS in order to assess the repeatability and validity of the data collection instrument, check the data collectors’ performance, highlight problems associated with the data collection tools, and ensure standardization of techniques. The collected data were checked for accuracy and completeness and collected on the day of collection before being filled. Data were coded and edited properly by the principal investigator before data entry. Before data analysis, data were cleaned, and 5% of the data were re-entered to ensure data quality.

Researcher-developed questionnaires were used in addition to the collection of demographic, dietary, lifestyle, and anthropometric data. Demographic information collected included marital status, place of residence, college type, college level, and selected personal lifestyle habits (smoking and physical activity). Physical activity was assessed using the International Physical Activity Questionnaire. The reliability and validity of the instrument have been established in previous studies [46,47,48]. In this section, participants were required to recall the type, frequency (days per week), and duration (hours and minutes per day) of each physical activity they performed during the last seven days. The assessment is based on the intensity of physical activities classified as vigorous (e.g., aerobic walking, jogging, and running), moderate (e.g., brisk walking, general home exercises, recreational swimming), and just normal walking. The level of physical activities is categorized as low, moderate, and high based on metabolic energy (metabolic equivalent of task, MET)-minute per week. The MET for walking is 3.3, for moderate activity is 4.0, and for vigorous activity is 8.0.

A qualitative food frequency questionnaire (FFQ) for specific food groups/items was also used. The FFQ included foods with plausible effects on PMS as reported by Cheng et al. (2013) [22], and other published research [26,27,28,29,30,31,32,33,34,35,36,37,38,39]. These included: starchy foods (e.g., bread, rice, pasta, pastries), milk and dairy products, caffeinated beverages (e.g., coffee, tea, energy drinks), leafy green vegetables (e.g., parsley, coriander, spinach, collard, Swiss chard), cruciferous vegetables (e.g., broccoli, cabbage, cauliflower, Brussels sprouts), other non-starchy vegetables (e.g., tomato, cucumber, bell peppers, green beans), fruit (e.g., bananas, apples, citrus fruit, melons, grapes), animal foods (e.g., red meats, poultry, fish, shrimp), herbal teas (e.g., cinnamon, black and green tea, sage, peppermint, thyme, ginger, chamomile) and high calorie/fat/sugar/salt foods that contribute high calories but have little nutritional value, e.g., high fat, sugar, and/or salt foods, fried foods, high-fat dairy products, eggs, refined grains, potatoes, corn and high-fructose corn syrup, and high-sugar drinks). Lifestyle factors, including smoking (cigarettes or *shisha*/hookah) and the duration of physical activity, frequency and type (from light to intense) were investigated, with examples provided for each activity level.

Anthropometric measurements were taken for each participant, including height (measured to the nearest 0.01 m) using a stadiometer (Seca 220, Hamburg/Germany). Body composition analysis was performed using a bioelectrical impedance (BIA) technique (InBody 230 model: MW160, Seoul/Korea) that measured body weight (to the nearest 0.1 kg), fat mass and body fat percentage (BFP), and visceral fat rating. Body mass index (BMI, kg/m^2^) was calculated and classified according to World Health Organization criteria, where participants with a BMI below 25.0 kg/m^2^ were considered as being normal weight, and those with a BMI of ≥25 kg/m^2^ considered as overweight or obese [49]. Anthropometric measurements were undertaken according to Centers for Disease Control and Prevention. National health and nutrition examination survey (NHANES): Anthropometry procedures manual.

BIA measurements were performed according to the manufacturer’s manual. Measurements were done for each student before eating breakfast and after removing any metallic accessories. Married participants were asked whether they were pregnant. All participants were asked about the presence of metal and pacemaker implants before starting BIA testing. 

PMS symptoms were measured using the Arabic Premenstrual Syndrome Scale (APMSS). APMSS is a newly developed scale to screen for PMS in Arabic women. Algahtani and Jahrami developed the scale [50] based on the criteria proposed by the Diagnostic and Statistical Manual of Mental Disorders, fourth edition (DSM-IV) [51]. The APMSS translates DSM-IV criteria into a four-point Likert-type scale with degrees of severity (none, mild, moderate, and severe). Operational definitions for mild, moderate, and severe PMS were: mild PMS symptoms do not interfere with the daily routine, moderate PMS symptoms interfere moderately with the daily routine, and severe PMS symptoms impair the daily routine [51].

The APMSS asks participants if they experienced specific PMS symptoms (from 1 week before menses to a few days into menstruation) in the past three months. The questionnaire contains 23 items in three domains: psychological symptoms, physical symptoms, and behavioral symptoms. Psychological symptoms include depressed mood, hopelessness, guilt feelings, anxiety/worry, affective labiality, increased sensitivity toward others, angry feelings, easily irritated/agitated, lack of interest, difficulty concentrating, loss of control, and feeling overwhelmed. Physical symptoms include lethargy/fatigue/decreased energy, increased appetite, cravings for certain foods, hypersomnia, insomnia, breast tenderness, breast engorgement or weight gain, headache, muscle/joint/back pain, and acne. Behavioral symptoms include symptoms that interfere with daily life routines, such as relationships, work, or school. The APMSS took about fifteen minutes to complete and about five minutes to score and interpret.

Pilot data testing was performed before the study started in order to assess the validity and repeatability of the questionnaire, address problems associated with the data collection tools, check data collectors’ performance, and ensure techniques were standardized. Cronbach’s alpha coefficients were greater than 0.8 for the APMSS. 

### 2.4. Data Analysis

The collected data were reviewed, double-checked for completeness and accuracy, and corrected daily before being entered into the spreadsheet. Random questionnaires were checked with entered values to control the quality of the data entry process. Data were cleaned before analysis to ensure data quality. To enhance the quality control for data management, data were checked for missing data, tested for the normality, and checked for outliers. Cronbach’s alpha was used to estimate the reliability coefficient properties of the PMS scale and the three groups of symptoms. A factor analysis of the 25 rating items in the PMS questions was conducted using the 300 cases. There are several suggestions about the ratio of cases or participants to items or variables; these range between 2:1 and 10:1. Thus, before running the factor analysis procedure, the Kaiser–Meyer–Olkin (KMO) Measure of Sampling Adequacy (MSA) and Bartlett’s Test of Sphericity were studied to mathematically establish suitability for conducting a factor analysis. The MSA index in our study was 0.9 (reference range 0 to 1.0). Additionally, Bartlett’s Test of Sphericity revealed highly statistical results of *p* = 0.001 indicating that there is a high probability of significant relationships between the variables. Procedure factor analysis that is exploratory in nature was carried out on the questions using the Maximum Likelihood Extraction (MLE) technique. The MLE method was used because of its known sensitivity in factor extraction as is produces parameter estimates that are most likely to have produced the observed correlation matrix. Promax rotation was used to ease the interpretation of the solution; the oblique type rotation was used to allow the factors to be correlated with each other. Three factors were extracted that have Eigenvalues of approximately 9.0, which explains about 40% of the total variance. The results of the exploratory factor analysis confirmed that grouping the PMS into psychological symptoms, physical symptoms and functioning symptoms, was rigorous and sound. These symptoms can be expressed as factor one, factor two and factor three. These factors also serve as an approach to both construct (within factors) and discriminant (between factors) validity. 

Analyses were reported based on the Strengthening the Reporting of Observational Studies in Epidemiology (STROBE) guidelines [52]. Questionnaires were coded, entered, and analyzed using Stata 13.1. Descriptive statistics, including mean, standard deviation (SD), and frequencies were calculated as point estimates for the sample. Also, 95% Confidence Intervals (CIs) were calculated to present an estimate of the variation around the point estimates. 

Multiple logistic regression analyses were used to examine the associations between demographic, dietary, and lifestyle factors (as exposures) and PMS symptoms (as outcomes). Odds ratios (OR) and 95% CI values were reported as measures of association between demographic characteristics (e.g., marital status), dietary factors (e.g., fruit, high calorie/fat/sugar/salt foods, and animal foods), and lifestyle behaviours (smoking and physical exercise) as exposures and PMS symptoms as the outcome.

## 3. Results

In total, 300 female students (3.4% of total population) enrolled in the survey and completed the APMSS. Participants’ ages ranged from 18–24 years (mean 20.07 years, SD 1.53 years). The mean ± SD BMI was 23.21 ± 4.41 kg/m^2,^ and body fat percent (BFP) was 34.87% ± 7.74%. The mean visceral fat rating was about 8 (7.9 ± 2.79) (Table 1). The vast majority (98.0%) of participants were single. More than half (about 58%) of the participants were in their second and third year, with a similar number from medical and health colleges, while around one-third (29.3%) were from basic and applied for sciences colleges. About three-quarters (about 75%) were living with their families and the remainder in university dorms (Table 1). Also, 87% of participants reported they were non-smokers. The vast majority of the participants did low intensity exercises (93.5%), while only 22% and 69% of the participants did vigorous and moderate exercises, respectively (Table 2).

Most participants (88.9%) had experienced dietary changes during pre-menstruation, with consumption of sweets (e.g., chocolate, cake, traditional eastern sweets such as *Kunafa* and *Baklava*) being the most pronounced dietary change. Frequent consumption of herbal teas and hot beverages during PMS was reported by 68.3% of participants, with cinnamon, green, and mint teas being the most common choices. Daily consumption of starchy foods, non-leafy, and non-cruciferous ordinary vegetables, and fruit was reported by more than half of participants; with weekly consumption of milk and leafy green vegetables being the most frequently reported choices. The majority of participants reported not using dietary supplements (ω-3 fatty acids and multi-nutrients) and consuming high calorie/fat/sugar/salt foods and animal foods (Table 2). The Cronbach’s alpha as a measure of internal consistency revealed that overall the PMS scale is 0.91 (91%), which is consistent in measuring the PMS. The results of the symptoms ranged between 0.7–0.9, suggesting that the scale is reliable, both in total and as a breakdown of symptoms. 

Generally, mild premenstrual symptoms were the most frequently reported, followed by moderate symptoms. Severe symptoms were the least frequently reported. Overall, the most frequently reported premenstrual symptoms were depressed mood (95%), lethargy/fatigue/decreased energy (92%), muscle, joint, abdominal and back pain (89.3%), feelings of anger (85.7%) and craving for certain foods (84.7%). The most frequently reported severe physical symptom was muscle, joint, abdominal and back pain (29.3%). Anger feelings (25%) and affective labiality (23%) were the most frequently reported severe psychological symptoms. The most frequently reported moderate symptoms were lethargy/fatigue/decreased energy (35%), depressed mood and lack of interest (33.3% each), and anger feelings (32.7%). Depressed mood (43.3%), difficulty concentrating (39.3%), and headache (38.7%) were the most frequently reported mild symptoms (Table 3). 

The multiple logistic regression analysis showed that smoking status was associated with increased risk of reporting psychological symptoms (OR 2.5, 95% CI 1.1–5.8; *p* < 0.05) and behavioral symptoms (OR 2.2, 95% CI 1.0–4.9; *p* < 0.05), while high calorie/fat/sugar/salt foods intake was associated with increased risk of reporting physical symptoms (OR 3.2, 95% CI 1.4–7.3; *p* < 0.05). However, fruit consumption (OR 0.34, 95% CI 0.125–0.92; *p* < 0.05) was associated with a decreased risk of reporting behavioral symptoms. Some variables showed trends, but did not reach statistical significance; for example, the association of herbal teas with psychological symptoms, moderate-intensity physical activity with functional impairment, and the intake of fruit and herbal teas with physical symptoms (Table 4).

## 4. Discussion

The present study aimed to assess the prevalence and severity of PMS symptoms among a sample of university students, to identify associated dietary and lifestyle factors, and clarify the association between PMS and BFP or BMI. To our knowledge, and according to the available literature, this is the first study that examined the prevalence of PMS among college students in the GCC countries, and the first one that examined its correlation with variable dietary and lifestyle behaviors. 

The average BMI of participating women was in the normal range. However, the BFP was higher than the acceptable normal range of 21–33% for women aged 20–39 years [53]. Nonetheless, the average visceral fat rating was lower than the BIA unhealthy cut-off value of 13. A value of 13 is equivalent to 130 cm^2^ visceral fat area, above which the visceral fat area is considered high and associated with increased risk for cardiovascular events [54]. However, no significant associations were found between anthropometric factors and PMS prevalence and symptoms. This finding is in line with the findings of Sadler and colleagues in a cross-sectional study on 974 women in the UK [43] and with the recent finding of Isgin-Atici and colleagues who found no significant differences in anthropometric measurements between PMS cases and their counterpart controls in Turkey [31]. However, this contradicts reports in Pakistani, Korean, Iran, and US adult females, in which high BMI, body fat and visceral fat were risk factors for reporting the prevalence and severity of PMS [55,56,57,58] and menstrual irregularity [59]. Nonetheless, this can be explained by the fact that the average BMI for our participants was within the normal range, with the least number of participants having a high BMI.

In our study, 95% of the participating young women experienced at least one PMS symptom, with varying degrees of severity. This was similar to a study by Tschudin and colleagues [60], that found 91% of participants reported at least one PMS symptom. When DSM-IV criteria were used, 35.3% of participants in the present study suffered from PMS. Muscle/joint/back pain and feeling angry were the most prevalent severe symptoms reported in the present study. The least common severe symptoms were behavioral symptoms (specifically, PMS symptoms interfering with work/school). Breast tenderness/pain was among the least common physical symptoms. These findings support other studies that found breast pain/tenderness was the least common symptom [50,61] while contradicting reports that breast tenderness/pain was among the most frequently reported symptom for women with PMS [11]. The low reporting of the somatic symptom of breast tenderness/pain in the present study may be explained by the fact that somatic symptoms tend to be associated with increased levels of inflammation [62]. 

Gold and colleagues [62] reported that premenstrual mood symptoms, abdominal cramps/back pain, appetite cravings/weight gain/bloating, and breast tenderness/pain appeared to be significantly and positively related to elevated *hs*-CRP levels, a biomarker of inflammation. However, information about levels of inflammation were lacking in the present study, meaning that we cannot confirm this explanation. Further research should be performed in this context. Abdominal pain/discomfort is one of the most frequently reported physical symptoms among surveyed females in the current work goes in line with the vast majority of surveyed females in many other works [6,40,41,63,64,65]. However, this finding disagrees with recent studies on relevant college students from China [30] and higher school girls in India [66]. This variation in the type of reported physical symptoms could be explained by the variation in dietary and lifestyle behaviors, and the coping practices that surveyed females were following before and during their menstruation. 

In the present study, consumption of starchy foods rich in complex carbohydrates did not show a significant association with PMS symptoms. This finding is consistent with a recent report by Houghton and colleagues that carbohydrates and fiber consumption was not associated with risk for PMS [67]. However, Hussein and colleagues [68] showed that a high intake of carbohydrates was associated with premenstrual symptoms (e.g., impaired concentration, behavioral change, autonomic reaction, and water retention). More preference has been directed toward whole grains in place of refined grains, where daily consumption of whole grains contributed to improvement in PMS symptoms in an open-labeled parallel randomized controlled trial [69].

Many studies have reported an association of smoking with increased premenstrual symptoms [13,70,71] and menstrual irregularity [59], other menstrual problems and miscarriage [72] with smoking for 5 or more years was associated with an increased prevalence of such symptoms. Moreover, one study showed that smoking status and depressive symptoms/anxiety were related to a higher menstrual symptom questionnaire score [70]. Another study revealed that the relative risk for PMS was 2.53 (95% CI: 1.70–3.76) for women who started smoking before age 15 years, when compared with those who had never been smokers [71]. Findings in the present study are consistent with these two previous studies, suggesting that adult female smokers have a higher risk of reporting PMS with more severe symptoms than non-smokers. This could be explained by the effect of cigarette smoking on dysregulating estrogen, progesterone, androgen, and gonadotropin levels, which may be involved in the etiology of PMS [71]. However, it is not guaranteed that smoking is directly involved in the etiopathogenesis of PMS or whether women experiencing PMS smoke as a means of alleviating their symptoms [71]. This may inform healthcare providers and decision makers in providing additional incentives for young women to avoid cigarette smoking, and consequently, to reduce the risk for PMS and its adverse health, social, and economic consequences. The lack of significant association between PMS symptoms and physical exercise is consistent with the finding of Sadler and colleagues’ study in the UK [43].

Fruit and vegetables are foods high in fiber, bioactive phytochemicals, and antioxidants [73]. In the present study, consumption of fruit appeared to be protective against psychological, physical, and overall PMS symptoms. Several previous authors have reported fruit, as part of the healthy Mediterranean diet, reduced the occurrence and severity of premenstrual pain and PMS symptoms [17,74]. Furthermore, the antioxidant power of various fruits may explain the protective role of fruit in PMS. Increased oxidative stress and reduced antioxidant capacity may occur in PMS, and an imbalance of oxidant/antioxidant systems may be a cause or consequence of various stress symptoms in PMS [75]. Daily consumption of fruit and non-starchy vegetables, as reported by more than half of the participants in the present study, might help to explain the low rates of severe PMS symptoms. This might be attributable to the anti-oxidant and anti-inflammatory agents provided by these plant foods, assuming that the onset and severity of symptoms are associated with elevated levels of serum inflammatory markers, including interleukin (IL)-2, IL-4, IL-10, IL-12 and interferon-γ [62,76]. 

The positive association between consumption of high calorie/fat/sugar/salt foods and psychological symptoms in our study is consistent with a study in India that reported a significant association between frequent consumption of high calorie/fat/sugar/salt foods and PMS symptoms (*p* = 0.004) [77]. Reducing consumption of high-fat high calorie/fat/sugar/salt foods may be associated with decreased PMS symptoms by converting estrogen into its inactive form. This was confirmed by recent work from Turkey and Iran, where adolescent girls with PMS reported higher intake of high energy and low nutrient-density foods [31,78]. Further, previous studies reported that female participants with PMS consume fast foods rich in sugar and total fat, caffeinated beverages, snacks, red and fatty meats in higher amounts and more frequently, while rarely consuming dairy products [17,22,79]. Nonetheless, Houghton and colleagues revealed that fat intake was not associated with higher PMS risk, and that high intake of stearic acid may be associated with a lower risk of reporting PMS [80]. The reported finding that women with PMS showed a significant increase in total energy and all macronutrients pre-menstrually when compared to nutrient intake post-menstrually [81] makes it difficult to ascertain whether increased caloric and macronutrient intake during pre-menstruation is a co-existing condition or as a result of PMS and its associated hormonal changes.

During the luteal phase and a few days into menstruation, many females crave certain foods [82]. In the present study, craving for sweets such as chocolate, cake, Eastern sweets (*Kunafa* and *Baklava*) was commonly reported by study participants (about 71%). This is consistent with research on food craving during the menstrual cycle, with foods rich in sugar, fat, and salt, such as snacks, chocolate, pastries, and desserts were higher during the premenstrual period [83,84]. 

The lack of a significant association between caffeine intake and PMS symptoms in the present study is consistent with the recent finding that caffeine and coffee intake is not associated with PMS [85]. A previous study found an OR of 0.79 when comparing women with the highest and lowest caffeine intakes (543 vs. 18 mg caffeine/day); even high caffeinated coffee intake was not associated with risk for PMS or any specific symptoms such as breast tenderness [85]. Conversely, Thu and colleagues [65] showed that PMS prevalence and severity were associated with increased caffeine intake. 

The limitations of the present study include those inherent in any study using self-administered questionnaires; namely, misclassification of answers, whether for the consumption of some food items or any symptoms. The PMS subgroup might have been confounded by mismatching participants who had similar discomforts in other phases of the menstrual syndrome. Further, the methods of selecting the sample of the present study means the results cannot be generalized to the overall university student population, or to adult females in the UAE, and is limited to college students, aged around 20, with a normal BMI, living with parents, who are not representative of all women in UAE. However, the UOS students in this study represented a heterogeneous population from diversified socioeconomic and family backgrounds across the seven UAE emirates. Also, the cross-sectional nature of this study restricts the ability to infer causation. The use of retrospective questionnaires is not the best method to collect data on PMS symptoms. The prospective methodology could be more fitting and compatible with the study objectives. Other limitations include the potential for uncontrolled residual confounding, lack of ability to assess temporal relationships when mentioning causal associations (e.g., it was unclear if participants were using these factors as a treatment for PMS symptoms or if the symptoms occurred after exposure to the factors), and lack of use of standard instruments. Another limitation is that standard instruments were not used for collecting data on dietary and lifestyle factors which could have resulted, not only in misclassification of this information but also in a lack of comparability of results to those from prior studies that have used standard instruments for collecting such data. The last limitation is that the authors were not able to discuss oral contraceptive use, which may have an influence on the presence of PMS, due to the format of exogenous hormones in oral contraceptives.

## 5. Conclusions

This is the first study investigating PMS among female university students in UAE and highlighting the high prevalence rate of PMS in this population. Interestingly, the results showed a significant association between the severity of PMS and dietary habits and lifestyle factors (smoking and high calorie/fat/sugar/salt foods consumption), while fruit consumption was found to be protective against PMS. The present study showed no significant correlation between the severity of PMS and anthropometric variables. There is a need for better detection and management of PMS among female university students so that they will not feel reluctant to seek proper medical advice. Therefore, designing educational programs should be tailored to increase awareness regarding the protective dietary and lifestyle habits and risky behaviors which influences PMS symptoms among female university students is warranted. Further research should be directed from the overall findings toward examining the hormonal, molecular and genetics changes associated with PMS among the UAE colleges and older female communities.

## Figures and Tables

**Table 1:** Participants’ sociodemographic characteristics and anthropometric measurements (*n* = 300).

Characteristics	
Age and anthropometrics (Mean ± SD *)	
Age (years)	20.07 ± 1.53
Height (cm)	160.8 ± 6.0
Weight (kg)	60.1 ± 12.7
BMI (kg/m^2^) **	23.21 ± 4.41
Body fat mass (kg)	22.30 ± 12.81
Body fat percent (BFP%)	34.87 ± 7.74
Visceral fat rating	7.9 ± 2.79
Sociodemographic characteristics (*n*, %)	
Marital Status	
Single	294 (98)
Married	6 (2.0)
College	
Basic and Applied Sciences	88 (29.3)
Medical and Health Sciences	173 (57.7)
Human and Social Sciences	39 (13.0)
College level	
First	52 (17.3)
Second	102 (34.0)
Third	71 (23.7)
Fourth	66 (22.0)
Fifth	9 (3.0)
Place of residence	
Family home	225 (75.0)
University dorms	75 (25.0)

* SD, standard deviation; ** BMI, body mass index.

**Table 2:** Participants’ dietary and lifestyle characteristics (*n* = 300).

Dietary and Lifestyle Characteristics	*n* (%)
Lifestyle behaviors	
Smoking	
Current smoker	39 (13)
Non-smoker	261 (87)
Physical activity (PA)	
Vigorous PA	66 (22)
Moderate PA	207 (69)
Light PA	280 (93)
Dietary behaviors before/during PMS	
Dietary changes during PMS	
No change	33 (11.1)
Craving sweets (chocolate, cake, Eastern sweets)	214 (71.3)
Craving savory snacks (nuts, potato chips, pickles)	31 (10.3)
Craving pastries (pizza, croissants, pies)	22 (7.3)
Use of herbal teas during PMS	
Cinnamon	78 (26)
Green tea	54 (18)
Mint	22 (7.3)
Chamomile	13 (4.3)
Ginger	12 (4.0)
Sage	12 (4.0)
Others (black tea, thyme)	12 (4.0)
Not consumed	95 (31.7)
Daily dietary behaviors	
Starchy foods	
1–2 times a day	171 (57)
2–4 times a week	118 (39.3)
2–4 times a month	11 (3.7)
Milk	
No intake	70 (23.3)
1–2 times a day	86 (28.7)
2–4 times a week	96 (32)
2–4 times a month	48 (16)
Dairy products	
No intake	12 (4)
1–2 times a day	139 (46.3)
2–4 times a week	124 (41.3)
2–4 times a month	25 (8.3)
Caffeinated beverages	
No intake	43 (14.3)
1–2 times a day	102 (34.0)
2–4 times a week	87 (29.0)
2–4 times a month	68 (22.7)
Leafy green vegetables	
No intake	13 (4.3)
1–2 times a day	85 (28.3)
2–4 times a week	149 (49.7)
2–4 times a month	33 (11.0)
Other	20 (6.7)
Cruciferous vegetables	
No intake	41 (13.7)
1–2 times a day	19 (6.3)
2–4 times a week	97 (32.3)
2–4 times a month	97 (32.3)
Other	46 (15.3)
Other vegetables	
No intake	24 (8.0)
1–2 times a day	185 (61.7)
2–4 times a week	70 (23.3)
2–4 times a month	15 (5.0)
Other	6 (2.0)
Fruit	
No intake	31 (10.3)
1–2 times a day	188 (62.7)
2–4 times a week	52 (17.3)
2–4 times a month	26 (8.7)
Other	0 (0)
Animal foods (meat, fish, poultry)	
No	14 (4.7)
Yes	286 (95.3)
High calorie/fat/sugar/salt foods	
No	31 (10.3)
Yes	269 (89.7)
Fish oil supplements (ω-3 FA)	
No	248 (82.7)
Yes	52 (17.3)
Multi-nutrient supplements	
No	236 (78.7)
Yes	64 (21.3)

ω-3 FA, omega-3 fatty acids; PA, physical activity level; PMS, premenstrual syndrome.

**Table 3:** Prevalence of premenstrual syndrome symptoms by the level of severity (*n* = 300).

Symptom	None	Mild	Moderate	Severe	Total
			*n* (%)		
	Psychological symptoms				
Depressed mood	15 (5.0)	130 (43.3)	100 (33.3)	55(18.3)	285 (95.0)
Hopelessness	95(31.7)	107 (35.7)	73 (24.3)	25 (8.3)	205 (68.3)
Guilt feeling	184 (61.3)	71 (23.7)	33 (11.0)	12 (4.0)	116 (38.7)
Anxiety/worry	74 (24.7)	106 (35.3)	72 (24.0)	48 (16.0)	226 (75.3)
Affective labiality	56 (18.7)	97 (32.3)	78 (26.0)	69 (23.0)	244 (81.30)
Increased sensitivity toward others	77 (25.7)	74 (24.7)	92 (30.7)	57 (19.0)	223 (74.3)
Anger feelings	43 (14.3)	84 (28.0)	98 (32.7)	75 (25.0)	257 (85.7)
Easily irritated/agitated	60 (20.0)	97 (32.3)	81 (27.0)	62 (20.7)	240 (80.0)
Lack of interest	61 (20.3)	94 (31.3)	100 (33.3)	45 (15.0)	239 (79.7)
Difficulty concentrating	118 (39.3)	118 (39.3)	48 (16.0)	16 (5.3)	182 (60.7)
Loss of control	84 (28.0)	96 (32.0)	78 (26.0)	42 (14.0)	216 (72.0)
Feeling overwhelmed	76 (25.3)	108 (36.0)	77 (25.7)	39 (13.0)	224 (76.7)
	Physical symptoms				
Lethargy/fatigue/decreased energy	24 (8.0)	108 (36.0)	105 (35.0)	63 (21.0)	276 (92.0)
Increased appetite	84 (28.0)	84 (28.0)	78 (26.0)	54 (18.0)	216 (72.0)
Craving certain foods	46 (15.3)	103 (34.3)	89 (29.7)	62 (20.7)	254 (84.7)
Hypersomnia	72 (24.0)	84 (28.0)	88 (29.3)	56 (18.7)	228 (76.0)
Insomnia	130 (43.3)	95 (31.7)	58 (19.3)	17 (5.7)	170 (56.7)
Breast tenderness	143 (44.7)	86 (28.7)	50 (16.7)	30 (10.0)	166 (55.3)
Breast engorgement or weight gain	142 (47.3)	79 (26.3)	52 (17.3)	27 (9.0)	158 (52.7)
Headache	103 (34.3)	116 (38.7)	51 (17.0)	30 (10.0)	197 (65.7)
Muscle, joint, abdominal and back pain	32 (10.7)	84 (28.0)	96 (32.0)	88 (29.3)	268 (89.3)
Acne	68 (22.7)	99 (33.0)	73 (24.3)	60 (20.0)	232 (77.3)
	Behavioral symptoms				
Symptoms interfering with:					
Relationships	156 (52.0)	95 (31.7)	35 (11.7)	14 (4.7)	144 (48)
Work or school	166 (55.3)	96 (32.0)	32 (10.7)	6 (2.0)	134 (44.7)
Daily routine	86 (28.7)	146 (48.7)	50 (16.7)	18 (6.0)	214 (71.3)
Cumulative psychological symptoms	1 (0.3)	118 (39.3)	142 (47.3)	39 (13.0)	299 (99.7)
Cumulative physical symptoms	2 (0.7)	99 (33.0)	173 (57.7)	26 (8.7)	298 (99.3)
Cumulative assessment of behavioral symptoms	67 (22.3)	167 (55.7)	60 (20.0)	6 (2.0)	233 (77.7)
Overall PMS		111 (37.0)	165 (55.0)	24 (8.0)	300 (100)

Total for mild, moderate, and severe symptoms only.

**Table 4:** Multiple regression analysis for dietary and lifestyle behaviors and associations with premenstrual syndrome.

Variable	OR	*p*-Value	95% CI
Physical symptoms			
Marital status	0.738	0.750	0.113–4.802
BMI	0.986	0.796	0.888–1.095
BFP	0.996	0.905	0.937–1.059
Caffeine intake	1.577	0.215	0.767–3.242
Smoking	1.548	0.312	0.664–3.608
Vigorous PA	0.985	0.961	0.527–1.840
Moderate PA	1.632	0.096	0.917–2.905
Light PA	0.734	0.575	0.249–2.162
Milk	1.599	0.145	0.849–3.011
Dairy products	0.657	0.570	0.154–2.800
Leafy green vegetables	2.199	0.268	0.545–8.877
Cruciferous vegetables	0.620	0.295	0.253–1.516
Other vegetables	1.312	0.668	0.379–4.533
Fruit	0.337	0.058	0.109–1.037
Animal foods	2.076	0.220	0.645–6.682
Fish oil (ω3 FA) supplements	0.750	0.428	0.368–1.528
Multi-nutrient supplements	1.658	0.154	0.827–3.323
High calorie/fat/sugar/salt foods	3.177	0.006 *	1.388–7.272
Herbal teas	1.709	0.058	0.982–2.977
Psychological symptoms			
Marital status	1.658	0.581	0.276–9.966
BMI	0.963	0.467	0.871–1.065
BFP	1.052	0.098	0.991–1.117
Caffeine intake	1.054	0.885	0.518–2.144
Smoking	2.545	0.027 *	1.11–5.829
Vigorous PA	0.882	0.678	0.489–1.593
Moderate PA	1.238	0.454	0.708–2.166
Light PA	1.280	0.638	0.458–3.581
Milk	0.979	0.945	0.532–1.800
Dairy products	0.756	0.711	0.172–3.314
Leafy green vegetables	0.547	0.428	0.123–2.434
Cruciferous vegetables	0.644	0.294	0.283–1.465
Other vegetables	1.006	0.992	0.308–3.289
Fruit	0.760	0.578	0.289–1.995
Animal foods	1.759	0.346	0.542–5.709
Fish oil (ω3 FA) supplements	0.942	0.864	0.478–1.857
Multi-nutrient supplements	0.909	0.770	0.481–1.718
High calorie/fat/sugar/salt foods	1.282	0.550	0.567–2.899
Herbal teas	1.705	0.051	0.998–2.912
Behavioral symptoms			
Marital status	1.658	0.581	0.276–9.966
BMI	1.023	0.699	0.911–1.149
BFP	0.995	0.887	0.927–1.068
Caffeine intake	1.939	0.212	0.686–5.479
Smoking	2.239	0.044 *	1.02–4.899
Vigorous PA	0.508	0.093	0.231–1.118
Moderate PA	1.995	0.065	0.957–4.159
Low PA	2.343	0.288	0.487–11.277
Milk	1.085	0.829	0.519–2.269
Dairy products	0.3153	0.107	0.077–1.282
Leafy green vegetables	0.615	0.587	0.106–3.558
Cruciferous vegetables	1.454	0.477	0.519–4.072
Other vegetables	2.485	0.266	0.499–12.358
Fruit	0.338	0.033 *	0.125–0.916
Animal foods	1.646	0.541	0.333–8.146
Fish oil (ω3 FA) supplements	0.982	0.965	0.437–2.207
Multi-nutrient supplements	0.702	0.373	0.323–1.527
High calorie/fat/sugar/salt foods	0.993	0.990	0.360–2.740
Herbal teas	1.113	0.753	0.570–2.170

* Significant at *p* < 0.05. BFP, Body fat percentage; BMI, Body mass index; CI, Confidence interval; ω-3 FA, Omega-3 fatty acids; PA, Physical activity level; OR, Odds ratio.

## Declarations

*Ethics approval and consent to participate:* All procedures performed in the present study were in accordance with the ethical standards of the institution (UOS Research Ethics Committee) and/or the national research committee, and the 1964 Helsinki declaration and its later amendments or comparable ethical standards. All participants signed an informed consent form before study enrollment.

## Abbreviations

APMSS	Arabic Premenstrual Syndrome Scale
BIA	Bioelectrical Impedance
BFP	Body Fat Percentage
BMI	Body Mass Index
CI	Confidence Interval
DSM–IV	Diagnostic and Statistical Manual of Mental Disorders, Fourth Edition
OR	Odds Ratio
PMS	Premenstrual Syndrome
UAE	United Arab Emirates
UOS	University of Sharjah.
